# Radiation Hardened LIDAR Sensor: Conceptual Design, Testing, and Performance Evaluation

**DOI:** 10.3390/s25237311

**Published:** 2025-12-01

**Authors:** Emil T. Jonasson, Christian Kuhlmann, Chris Wood, Robert Skilton

**Affiliations:** United Kingdom Atomic Energy Authority, Culham Campus, Abingdon OX14 3DB, UK; christian.kuhlmann@ukaea.uk (C.K.); chris.wood@ukaea.uk (C.W.); robert.skilton@ukaea.uk (R.S.)

**Keywords:** radiation, LIDAR, sensor, robotics, nuclear, GaN

## Abstract

In scenarios involving radiation such as decommissioning of nuclear disasters and operating nuclear power plants, it is necessary to perform tasks including maintenance, demolition, and inspection using robots in order to protect human workers from harm. LIDAR (LIght Detection And Ranging) sensors are used for many demanding real-time tasks in robotics such as obstacle avoidance, localisation, mapping, and navigation. Standard silicon-based electronics including LIDAR fail quickly in gamma radiation, however, high-radiation areas have a critical need for robotic maintenance to keep people safe. Sensors need to be developed, which can cope with this environment. A prototype including most required transmitter and receiver circuits is designed utilising components expected to provide up to (1 MGy) gamma radiation tolerance. Initial results testing the concepts of the laser transmission and detection in a lab environment shows reliable signal detection. Performance tests utilising multiple receivers show a linear relationship between receiver separation and measured time difference, allowing for the possibility of calibration of a sensor using the time difference between pulses. Future work (such as radiation testing trials) is discussed and defined. These results contribute to de-risking the feasibility of long-term deployment of LIDAR systems utilising these approaches into environments with high gamma dose rates, such as nuclear fission decommissioning, big science facilities such as the Large Hadron Collider, and remote maintenance systems used in future nuclear fusion power plants such as STEP and EU-DEMO.

## 1. Introduction

When conducting operations in environments with high levels of gamma radiation, such as in decommissioning of nuclear disasters and operating nuclear power plants, it is often necessary to perform tasks including maintenance, demolition, and inspection using robots to protect human workers from harm. The generation of power in both fission and fusion energy mainly involves the creation and utilisation of neutrons (either as a by-product of heavy atoms splitting or light atoms fusing together). These neutrons inevitably interact with materials inside the reactor such as nuclear vessel walls and shielding blankets. The resulting “activation” of these materials means that, even during maintenance shutdowns, the power plant environment will contain extremely high levels of gamma radiation. The standard silicon-based electronics fail quickly in gamma radiation, yet these are the areas most in need of robotic maintenance, and as such, robotic components such as sensors need to be developed, which can cope with this environment.

Robotic and remote systems rely to a large extent on non-contact ranged sensors for reliable and robust interaction with their environment. Common examples of sensors, which monitor the external environment and provide distance information to robotic systems include optical cameras (depth or stereo), infrared sensors, ultrasonic sensors, radar, and LIDAR. These all provide varying combinations of information about the external world such as distance to obstacles, colour, and movement speed of nearby objects. The challenge of producing radiation tolerant versions of these sensors varies depending on their specific construction, and significant progress has already been made on sensors such as cameras [1]. However, much work remains to create a full suite of non-contact ranged sensors, which can be deployed in a high radiation environment to enable robotic and automated operations.

Over the last 10–20 years, the explosive growth in capabilities and performance of mobile 2D and 3D-LIDAR (LIght Detection and Ranging) systems as well as their high degree of usefulness have led to increasing use in the field of robotics [2] to the point where it has become a standard sensor in robotics and remote applications. LIDAR is used for many demanding real-time tasks in robotics such as obstacle avoidance, localisation, mapping, and navigation. A large number of autonomous systems and robotics navigation and localisation algorithms (such as those used in self-driving cars) now heavily depend on LIDAR sensors [3], and this trend is expected to continue [4].

For future fusion power plants, cost-effective maintenance (combining reliability, speed, and the ability to continuously operate large numbers of inspection and maintenance systems in parallel across a large facility) will rely to a large extent on automation [5]. The suitability of LIDAR technology to support this automation in fusion remote maintenance contexts has previously been explored. It has been demonstrated that LIDAR technology is suitable for the measurement of distances between a robotic maintenance system and internal tokamak surfaces [6] and can produce accurate 3D models of the inside of a fusion vessel [7]. In addition, LIDAR has been used for external equipment inspection of plant components using a mobile robot [8]. However, these have only been demonstrated in environments with very low levels of radiation, and the previously demonstrated technologies are not compatible with environmental conditions in more extreme scenarios.

The LIDAR-based technologies will also be useful in more traditional nuclear facilities. In addition to the standard planned decommissioning of fission power plants, which have reached their natural end of life, there are more unplanned nuclear decommissioning programmes. These programmes include the Chernobyl and Fukushima Daiichi [9] reactors, which suffered catastrophic accidents in the past, as well as nuclear R&D sites such as at Sellafield in the UK and the Hanford site in the USA, which did not have a clear end-of-life plan when originally constructed. In addition, some “big science” facilities such as the Large-Hadron Collider, hosted by CERN, and the European Spallation Source in Lund, Sweden, also share the challenge of high gamma radiation doses created through activation of materials as part of the normal course of experiments. Many of these places share the challenge of poorly mapped, high gamma dose radioactive environments, sometimes with no up to date facility schematics or 3D CAD models. Human access to certain parts of these facilities can be impossible or intolerably risky. As such, operations are carried out in these areas using robots, and these stand to benefit significantly from the capabilities that are enabled through the use of radiation tolerant LIDAR sensors, especially when combined with other sensor modalities such as cameras and radar [10].

However, standard COTS LIDAR sensors are highly complex assemblies of silicon semiconductor components. There is a need for on-board silicon electronic components such as high-accuracy digital timers, laser diodes and detectors, angular sensors and motor drives, power regulation as well as on-board processing, and data buses for transferring results to a wider system. These types of components are generally very sensitive to the residual gamma radiation, which can be found in most nuclear environments, and start to fail at total integrated dose levels of around 100 Gy. Past deployments of mobile COTS (Commercial Off The Shelf) LIDAR devices into some radiation environments have been successful due to short deployments (minutes or hours) in environments of modest radiation dose of <1 Gy/h [6,7], hence not having time to accumulate a significant dose, which could cause premature failure. More extreme scenarios requiring total integrated dose tolerances of 100 Gy+ will need specialised LIDAR design approaches to ensure long term survivability.

One example of such a radiation-hardened LIDAR comes from the ITER programme. They have invested in the development of a “periscope”-style LIDAR sensor known as the IVVS, or the In-Vessel Viewing System, versions of which has been under development for over 20 years. The design of this system keeps the sensitive electronic control system outside the high-radiation area of the fusion vessel, and inserts only a probe (already demonstrated to be gamma tolerant to 4.8 MGy) containing piezoelectric motors, optical encoders, and various laser optics support equipment into the high radiation area [11]. The laser beam originally was aimed using a prism actuated by the piezoelectric motors, but later designs have replaced this with a mirror arrangement [12]. This system is expected to produce sub-millimetre accuracy component inspection [13]. The main limitation of this solution is its built-in and fixed nature, with portable applications being impossible without incorporating a bulky umbilical cable trailing back to a low-radiation area to house laser diodes, photon detectors, processing devices, and so on.

LIDAR devices adapted for different environments are available for purchase since many applications involve the use of these sensors in rain, snow, cold, or hot temperatures. However, to date no portable LIDAR sensor suitable for use in higher gamma radiation environments (dose rates of up to 1 kGy/h, total integrated dose between 10 kGy and 1 MGy) has been produced. This is likely due to the inherent challenge of implementing a radiation tolerant digital sensor, which traditionally is composed mainly of silicon-based components. However, in recent years, some key rad-hard LIDAR technology building blocks, such as the high-accuracy digital timer devices needed for accurate laser ranging [14], have been demonstrated, and as such it now appears feasible that a portable LIDAR scanner could be developed given investment of time and effort [15].

A fully portable 1 MGy rad-hard LIDAR device would represent a step change in capability for the fission and fusion programmes, and enable long-term deployment of this technology into extremely harsh environments, bringing many benefits. The goal of this work was to carry out a feasibility study to investigate whether a portable prototype 1D LIDAR design could be created using only electronic and optical components known or expected to have a radiation tolerance of around 1 MGy. Unlike previous approaches, this device would need to have its transmission and detection subsystems, timers, and power regulators as well as supporting components capable of being situated locally in the high radiation environment, instead of relying on fibres or waveguides to transmit the laser light into the work area. One way to accomplish this is by excluding standard silicon-based components known to be sensitive to gamma radiation and re-implementing their functionality using these alternative components. If successful, this would demonstrate the basic concept of a transmit-receive-time laser ranging device, with the further development of missing subsystems to be completed in future work.

The context and rationale for this development has been described in Section 1. Section 2 introduces the basic concept of LIDAR operation. Section 3 reviews relevant literature in the fields of radiation hardness and electronics/optics. Section 4 describes the design of the LIDAR prototype. Section 5 describes the tests carried out on the prototype, and Section 6 presents the results. Section 7 presents a discussion of the results and what they mean, and Section 8 provides the conclusions resulting from the work as well as future development plans.

## 2. LIDAR Principles of Operation

**Figure 1:**
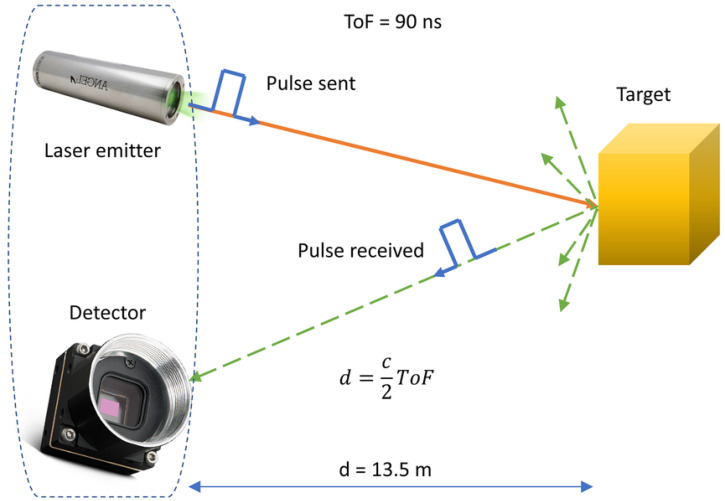
LIDAR example operation, from Ref. [16]. The general principle of a LIDAR is straightforward: a timer is started when a pulse is sent from a laser emitter, this signal bounces off an object in the world and returns to a detector. The timer is stopped and the distance to the object can be calculated using the known speed of light in the environment and some basic trigonometry.

At its most basic, the operating principle of a LIDAR is a simple one. A laser pulse is sent out, which travels at a known speed (the speed of light). This pulse then bounces off an object in front of the sensor, and a portion of the laser pulse then returns to the LIDAR to be collected by a receiver. The system measures the difference in time between the transmitted and the received pulses, and therefore the distance to the object in question can be calculated using the known speed of light in air (Figure 1). This technique is often referred to as Time-of-Flight (ToF) sensing. In order to improve accuracy, the effects of small delays in the transmitter circuit can be mitigated by adding a second receiver, which detects the actual transmission of the laser pulse, and uses this detection signal to start the digital timer. This operation results in a several required subsystems, further detailed in this section.

### 2.1. Transmitter

The role of the transmitter is to transmit a laser pulse of a suitable amplitude and width. This role is generally filled by a laser diode, which is pulsed using a signal generator. This pulse needs to be of an appropriate intensity to avoid excessive walk error and provide a suitable signal-to-noise ratio.

A laser diode is required to transmit concentrated light in a narrow spectral range. The narrow spectral range allows filters to be used in the receiver, only permitting the wavelengths of laser light to pass through. In doing so, this significantly reduces the signal-to-noise ratio with improvements of this ratio being related to optical power and width of spectral range. Generally, a higher permitted pulse power is favourable when selecting a laser diode.

A pulse generator is needed to be able to produce an optical pulse on demand, more specifically, a single high-power narrow pulse. An oscillator is one alternative, but these provide less control over when pulses are transmitted and as a result makes processing significantly more difficult.

In general, one-shot narrow pulse generators are triggered upon a reference start pulse and will be used to control a high-power laser driver, allowing large instantaneous peak laser power. The smaller the pulse is, the larger the instantaneous power can be without damaging the laser diode or equipment/people exposed to it. Additionally, shorter pulses lead to a reduced timing error in the system. A final requirement of these generators is that a clean pulse shape should be produced to allow for a well-defined laser pulse, which makes detection and processing easier.

### 2.2. Receiver

The receiver subsystem has the role of collecting the laser return signal and indicate to the timer circuit that this has occurred. In order to ensure an accurate pulse timing, a separate internal receiver is often used to detect the actual transmission of the laser pulse and use this to start the clock in the timer circuit.

The laser returns are detected by a photon detector such as an MPPC (Multi-Pixel Photon Counter), this detector is also known as a silicon photomultiplier (SiPM). It is a solid state photodetector that uses multiple avalanche photodiode (APD) pixels operating in Geiger mode [17]. APDs are a type of semiconductor photodiode detector and are highly sensitive to incident photons. These devices utilise the photoelectric effect to greatly amplify a small initial photonic signal and produce an output voltage proportional to the intensity of incident light. When operating in Geiger mode, these Single Photon Avalanche Diodes are very similar to their Avalanche Diode Counterpart with the key difference between the two being the level of reverse biasing used. Where APDs have a reverse bias below their reverse breakdown voltage, SPADs use reverse biases well above their reverse breakdown voltage. These devices exhibit sensitivities able to measure the arrival of a single photon and can be used to measure timing of photon receipt due to the high speed of avalanche formation and progression, and the low jitter associated with such devices.

MMPCs/SiPMs are constructed from a grid of SPADs with each pixel generally ranging from 10 to 100 micrometres in size. Each SPAD is operated in the Geiger mode and is equipped with a quench resistor to provide passive quenching to the system. Gains of these devices tend to be in the order of $10^{6}$, this is similar to that of photomultiplier tubes.

When a connected photo-receptive device such as an MPPC produces an output signal, the amplitude and pulse-width of the signal will generally be very small, and the frequency will generally be very high. As a result, high speed amplifiers must be used to greatly increase a received signal’s amplitude ready for discrimination. The output signal of an APD, SPAD, and SiPM are currents, hence the amplification is commonly achieved in one of two ways: (1) using a trans-impedance amplifier (TIA) or (2) using a resistor (as a form of trans-impedance amplifier) coupled with a high-speed gain stage.

### 2.3. Timer

The Time-to-Digital Converter (TDC) is the backbone of the processing system in leading-edge ToF and enables the timing of laser pulses transmitted and then received. A basic design involves the TDC receiving start and stop pulses from the transmitter and receiver discriminators following a pulse. The timing between these pulses is then calculated. Alternatively, a reference pulse can be used in tandem with the transmitter and receiver discriminators to help improve system efficacy and eliminate dead time at the start of some TDC’s ranges.

### 2.4. Logic

Once a laser pulse has been transmitted, received, and a time difference has been established, a logic circuit is necessary in order to mathematically compare the timestamps and calculate distance using the speed of light in air. This can either be completed outside of the radiation environment using long cables or other radiation hardened communications solutions, or within the radiation environment using specialised analogue/digital logic.

## 3. Radiation Effects on LIDAR Components and Mitigation Strategies

This section summarises existing literature regarding effects of gamma radiation on required LIDAR semiconductor components, as well as alternative materials and components for providing the same type of functionality but with a greater gamma resistance.

Silicon semiconductors have poor resilience to even relatively small (<1 kGy) ionising (gamma) radiation doses [18]. Ionising radiation causes failure of silicon devices due to charge build-up (especially in oxides) and an increase in density of surface states, resulting in multiple possible failure modes. These include a failure to switch states (for example, a switch being stuck on or off), an increase in delay times, and an introduction of a prohibitively large leakage current preventing the device from performing its intended function, which can all be summarised as the semiconductor failing to work as intended.

Even doses of several hundred Gys have been shown to induce failures in silicon-based power regulators, which rely on bipolar transistors for their core functionality [19]. There are several options for creating radiation-hardened semiconductors, from custom silicon-based implementations designed from the ground up to mitigate gamma radiation effects [14,20] to the utilisation of component structures, which are inherently more tolerant to gamma effects. In addition, the utilisation of wide bandgap (WBG) semiconductors such as silicon carbide (SiC) [21,22], gallium nitride (GaN) [23] and even diamond [24] can create more robust components due to their wider bandgap resulting in less electron mobility due to gamma as well as their inherent structures being less prone to the same charge build-up effects as seen in classic silicon [25]. As an example, gallium nitride has a bandgap of 3.2 electronvolts (eV), while silicon’s bandgap is only 1.1 eV—this enables GaN a larger breakdown voltage and more thermal stability at higher temperatures. It also has a higher breakdown field (3.3 MeV/cm vs. silicon’s 0.3 MeV/cm), which results in improved high voltage performance [26]. However, the manufacturing process is much less mature than that of silicon, resulting in higher costs. As such, only certain types of GaN devices have entered mainstream use, mainly in the area of power electronics.

In the area of LASER diodes, experimental testing has shown that indium-galluim nitride (InGaN) and aluminium tri-oxide (Al_2_O_3_/sapphire) photo-emissive devices offer significant benefits in irradiated environments [23]. InGaN laser diodes have been tested under 1.5 MGy gamma (total integrated dose) and showed only a 20% decrease in emissivity. Furthermore, annealing of these devices has shown to help reverse the effects of radiation damage on performance.

As for transistors, gallium nitride (GaN) has been shown to have excellent radiation resistance with HEMTs (High Electron Mobility Transistors) having been tested to high levels of gamma doses and shown to exhibit little to no degradation as well as general performance gains over silicon devices. Studies have shown very minor changes at doses up to 20 kGy gamma TID (Total Integrated Dose) [27], and component parameter changes have not significantly affected component performance at doses up to 2 MGy in other studies [28]. GaN has seen a significant amount of recent development, and many consumer devices now utilise this technology. This is due to its excellent performance such as very large switching capacities, low gate capacitance, and huge figure of merit compared to silicon components. This has allowed GaN to take a large hold in the power electronics industry, with GaN commonly used as a semiconductor switch in power supplies.

For photon detectors, one study has shown that Hamamatsu Multi-Pixel Photon Counter technology can perform in irradiated environments with predictable changes [29]. Results show decreasing effects after 100 kGy of Gamma (total integrated dose) implying that effects are plateauing at doses approaching 1 MGy. It is expected that a calibration system could be used to help negate these effects, especially increases in the Dark Count Rate (DCR) as this is the main affected parameter under gamma irradiation due to its dependence on charge trapping. Alternative wide bandgap technology-based solutions also exist but these are not yet easily available as COTS devices. SiC APDs have been investigated in the use of a LIDAR system [30], and InGa(N)P devices may also provide a solution [31].

Previous research has shown excellent results utilising custom-designed silicon Time-to-Digital Converters (TDCs) capable of withstanding 1 MGy TID [14]. The resulting COTS components based on this research are available for purchase from the MAGICS technologies NV https://www.magics.tech/ (accessed on 6 July 2025). The same manufacturer also provides MOSFET drivers with a total integrated dose tolerance of 1 MGy.

## 4. Prototype Design and Component Selection

In order to simplify the manufacture and testing, the prototype system targeted was a 1D LIDAR system. This restricted the design challenge to the proof of concept of LIDAR transmission and detection.

Various different radiation mitigation design approaches are generally used in the design of systems for radiation environments. The approach taken to radiation hardness in this project was to use a mix of commercially available radiation-hardened silicon ASICs (Application Specific Integrated Circuits) and wide band gap intrinsically radiation tolerant semiconductor components such as GaN FETs. The aim was to provide the same functionality as can be achieved with non-rad hard silicon COTS devices, but with a significantly increased gamma radiation tolerance.

**Figure 2:**
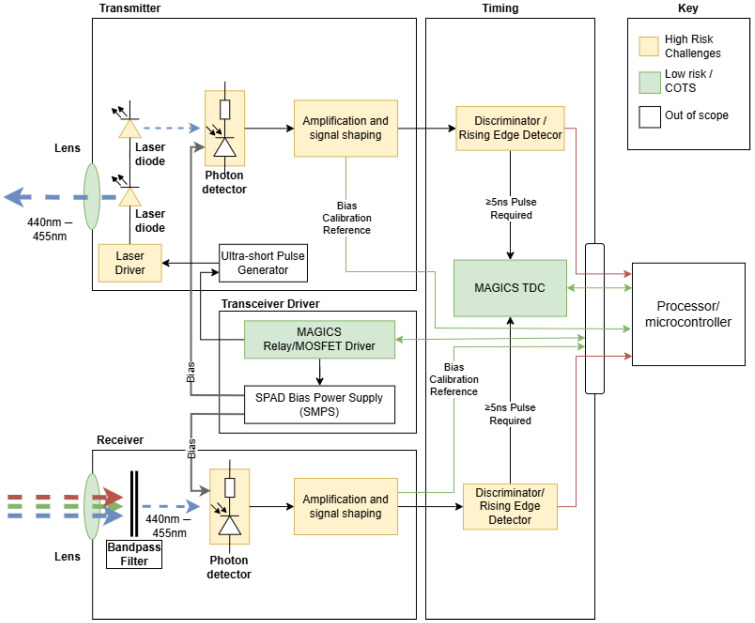
System design diagram outlining proposed design and challenges. This shows the full complement of necessary functionality for a portable 1D LIDAR demonstrator, and highlights in yellow the areas covered in this work (high-risk-challenges). The green areas are low risk components available as COTS and the white areas are components that are out of scope of this particular work.

The high-level design was inspired by the structure of the 1D-LIDAR demonstrator SECO-RANGEFINDER-GEVK, manufactured by OnSemi (ON Semiconductor Corporation, Scottsdale, AZ, USA) and available as a COTS item along with full design documentation. A high-level design diagram was created, this is shown in Figure 2. As can be seen, whilst some items (lenses, time-to-digital converter, relay/MOSFET driver) are available as COTS items tolerant to 1 MGy TID, most of the design required novel implementations or utilising components not yet proved to be gamma radiation tolerant to 1 MGy. The key items needing investigation were the laser driver and laser diode, amplification/signal shaping circuits, photon detectors as well as discriminators/rising edge detectors. As such, the design efforts were focussed in these areas. The ultra-short pulse generation, on-board processing of timing data as well as the SPAD bias power supply were designated as out-of-scope of this work since these subsystems pose their own significant challenges requiring further development.

### 4.1. Transmitter

Critical to the transmitter circuit is the generation of short voltage/current pulses for driving the laser diode and sending out distinct pulses of laser light. Whilst creating a rad-hard pulse generator is out of the scope of this work, a high slew-rate circuit was designed to sharpen the pulse edge before feeding this edge into the COTS pulse generator.

**Figure 3:**
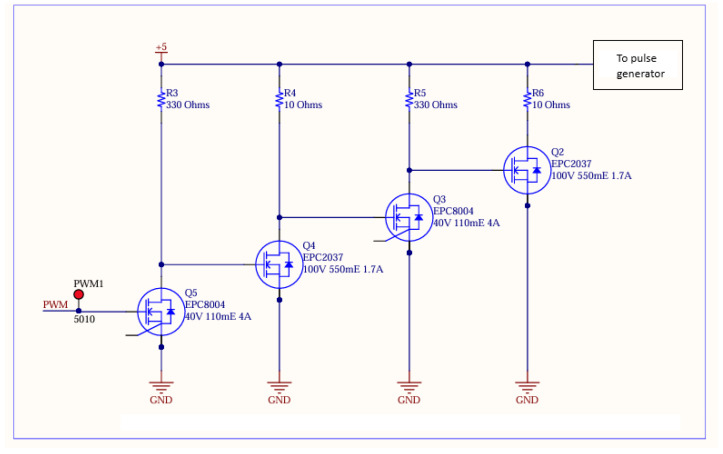
High slew-rate edge circuit designed for transmitter. This contains a set of cascaded NMOS inverters to sharpen the pulse edge. EPC8004 is used for strong pulldown and EPC2037 is used for rapid switch on/off. Varying resistances were used to optimise current draw.

**Figure 4:**
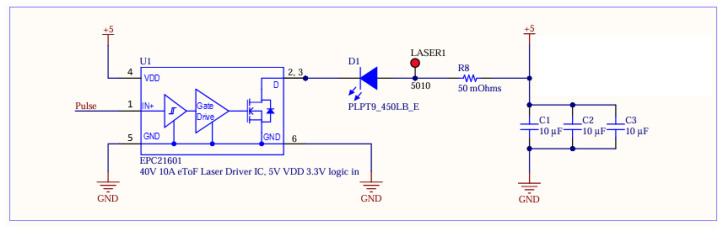
Laser driver designed for transmitter. The capacitor bank was used to facilitate 15A+ current draw of laser without supply interruptions.

Each transmitter board contains a set of the NMOS inverters to increase the slew rate/decrease the rise time of the input timing signal (Figure 3). This is then fed into a COTS narrow pulse generator and then finally fed into the laser driver IC EPC21601 (Figure 4). The full list of components can be found in Table 1.

### 4.2. Receiver

**Figure 5:**
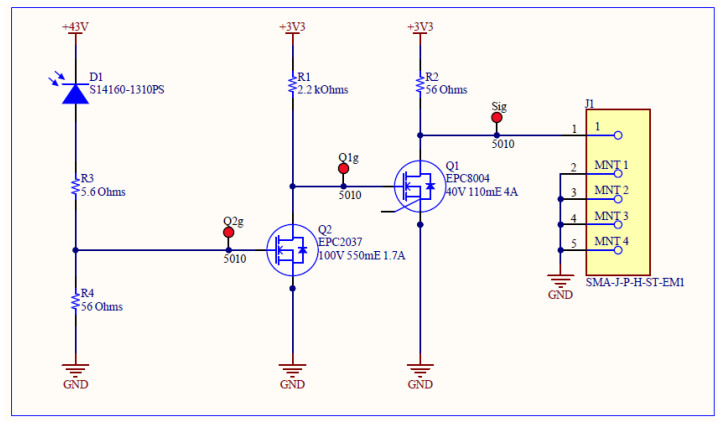
Receiver design. The MPPC is biased with a resistor providing trans-impedance gain. The output of this is then fed into an inverting discriminator (using an EPC2037 GaNFET) and then an inverter (EPC8004 GaNFET) to provide an active high signal.

The receiver board was created using the Hamamatsu MPPC S14160-131(0/5), since these devices were available as COTS components and have been previously radiation tested [29]. The expected radiation tolerance of 100 kGy–1 MGy, with the only degradation effect reported being a manageable parameter degradation, makes this a very interesting candidate for the system. The MPPC is biased with a resistor providing trans-impedance gain. The output of this is then fed into an inverting discriminator (using an EPC2037 GaNFET) and then an inverter (EPC8004 GaNFET) to provide an active high signal (Figure 5). These components were also chosen for their likely radiation tolerance. The complete component list can be found in Table 2.

### 4.3. Design Approach

**Figure 6:**
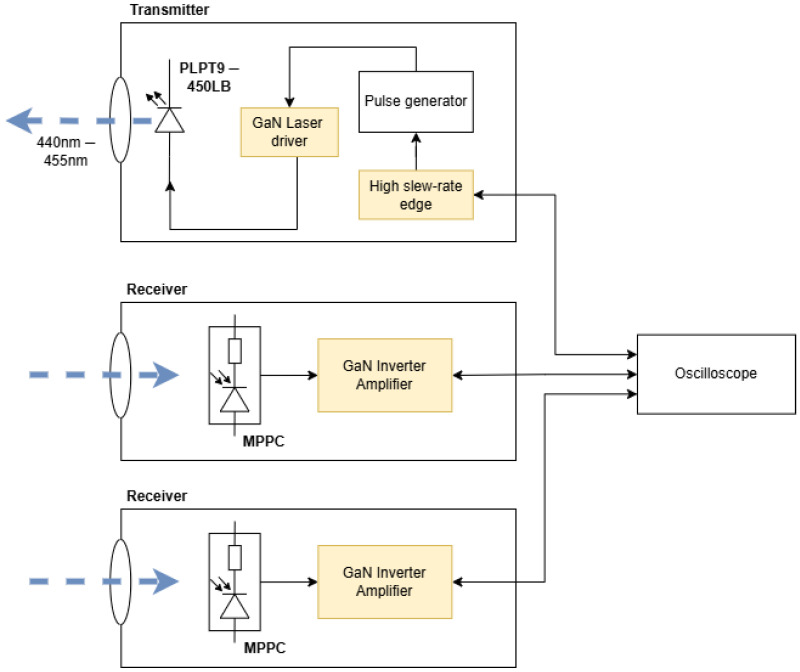
High-level diagram of the test system for initial LIDAR transmitter and receiver.

To enable more straightforward testing, the subsystems were designed as separate boards to isolate the functions of the system and to enable more straightforward testing and mitigation of errors. This approach can be seen in Figure 6.

Expected radiation tolerance of the overall resulting device is expected to approach 1 MGy gamma total integrated dose. All components used (Table 1 and Table 2) are either passive components with limited radiation sensitivity, active components chosen due to their material having natural radiation resistance, or silicon components expected to operate at very high doses with manageable performance degradation so as to not hinder operations.

## 5. Testing and Performance Evaluation

Boards were assembled and electrically tested. The testing was conducted inside an interlocked light-tight box, helping to shut out ambient light to enable testing of the transmitter and receiver without the need for an optical filter to remove all other wavelengths, along with increased eye safety. The transmitter and receiver circuits were operated as separate units using bench-top power supplies, and timing measurements were taken with a separate external oscilloscope able to measure and discriminate between nano-second time delays.

Initial experiments included a single transmitter and a single receiver, both installed in-line on movable T-nuts, which could be slid along a line using an aluminium extrusion, providing direct line of sight between the transmitter and receiver to prove the basic of operations without adding unnecessary complexity. This gave promising initial results, proving functionality in the simplest possible setup. The signal was registered as detected when a voltage level of 4 V was seen at the output of the Receiver circuit. The smallest distance between transmitter and receiver was 200 mm and the largest 400 mm (restricted by the size of the light safe test area).

**Figure 7:**
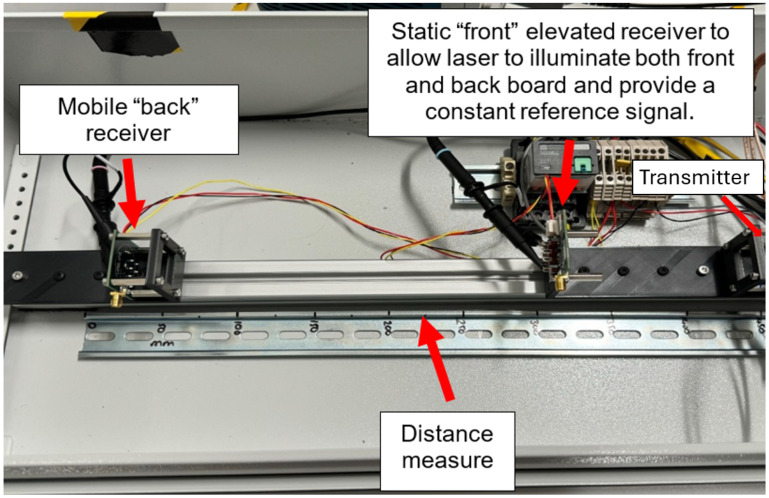
Test setup using a transmitter and multiple receivers. The receiver closest to the transmitter is stationary throughout testing and provides a reference signal. The back receiver moves along a labelled track to enable the setup to measure different distances and detection time delays between the transmitter and the two receivers.

A static, second receiver was then added to be used as a differential measurement sensor, in the same arrangement as that described in Figure 2. This was raised up to be out of the direct line of the laser yet still close enough to receive sufficient laser light to trigger the photon detector (Figure 7). To carry out a measurement, a setpoint was chosen near the start of the rising edge of the signal from the receiver circuit and timed between the reference receiver and target receiver to produce a result. In order to provide linear operation, the reference was chosen to be at the start of the rising edge as this section is least sensitive to noise and operating variations within the circuit. A range of different separation distances between the two Receiver boards were used. These were smaller than the distances achieved in the previous test since the “start” receiver was placed at 150 mm from the transmitter, and the “stop” receiver could only be placed a maximum of 225 mm away from this point due to the limited size of the test enclosure.

## 6. Results

**Figure 8:**
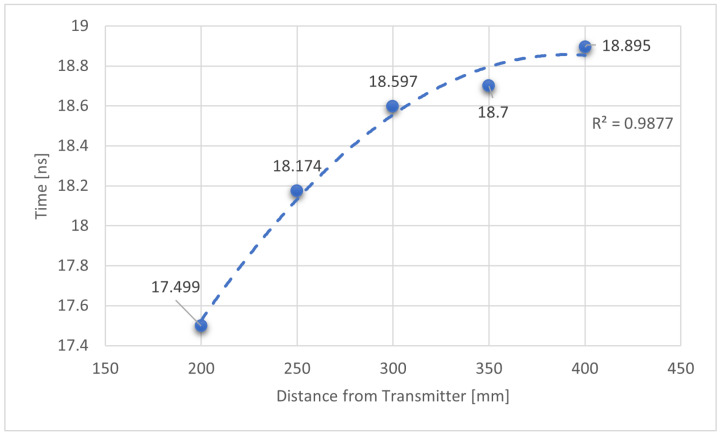
Plot showing the time difference in nano seconds between signal transmission and receiver detection during initial tests using a single receiver, showing reliable signal detection. Non-linear result likely caused by light intensity drop-off with distance and use of single receiver. Step size: 50 mm.

This section describes the results collected from the bench testing of the transmitter and receiver. Initial tests utilising only one transmitter and one receiver demonstrated successful basic operation, in that pulses were transmitted, clearly received and could be distinguished from the background noise (Figure 8). Non-linearities result from different rates of capacitor charging: the inverse square law relating optical intensity at receiver to the distance between the transmitter and receiver means that the intensity of light reaching a given sensor will be much higher closer to the transmitter, where the larger signal will result in a faster charging of the capacitor and the 4 V trigger level being reached quickly. The signal being inversely proportional to the square of the distance is therefore reflected in the results.

**Figure 9:**
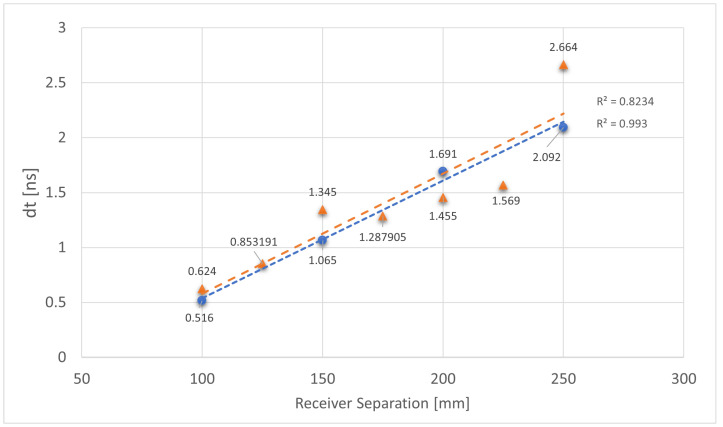
Plot showing the time difference in nano seconds and hence distance between both receivers arranged in a differential mode. Two different series of test at regularly increasing distances are plotted; Blue series has four data points at a step size of 50 mm, orange series has seven data points at a step size of 25 mm.

Differential data sets are shown in Figure 9. This shows a well-defined linear response, due to the use of the start of the rising edge on both receivers as trigger point for measurements.

As the target of this work was merely to prove the concept of a rad-hard LIDAR design, and since the test setup only allowed data to be collected over a small range, only a few trials were carried out to prove the basic performance of the sensor.

## 7. Discussion

To produce a rad-hard LIDAR solution, many electrical components and subsystems must be substituted with rad-hard counterparts creating a different set of design constraints and limitations. In order to transmit a laser pulse for use in distance measurement, a laser diode is required. This work has shown how an InGaN laser (likely to be very radiation tolerant) can be powered using mostly novel circuits specially designed to use other GaN components known for their radiation tolerance.

The receiver light sensor was chosen for its known radiation tolerance of 100 kGy from previous publications, as well as indications that the damage was plateauing at higher doses [29]. In order to use this sensor in the system, a set of discriminators were designed, again using components made from GaN material known to have a high gamma radiation resistance.

The results achieved in this work are very promising. Initial results testing the concepts of the laser transmission and detection showed reliable signal detection, even though the first set of distance measurements showed a non-linear result due to the side effects of using a single receiver and the trigger voltage being set to 4 V (this turned out to be too high). The performance tests, utilising multiple receivers with a much lower trigger voltage to identify the start of the pulse, showed a linear relationship between receiver separation and measured time difference. This allows possibility of calibration of a sensor using the time difference between pulses to determine separation between the local transmitter/receiver pair and the remote receiver. Lower trigger voltage reduces the effects of distance due to reduced charge requirements. Further testing with a larger test area, measuring greater distances, will further contribute to the confidence of this design approach.

Of course, a LIDAR works by locating both receivers on the sensor body itself and bouncing the signal off of an external object whose distance shall be measured. This technique is well known and used in all commercial off the shelf LIDAR devices, and whilst outside the scope of this initial work it is also a logical next step implying future work to prove that this technique can be replicated using this non-standard radiation hardened design. The main technical risk in this area is expected to be that the non-standard wavelength of InGaN laser diodes could affect the options for filtering the signal in the presence of other light and make the bounced signal harder to detect than expected.

A further consideration stemming from the use of a ∼450 nm laser is that this is in the visible light spectrum, and so it brings additional safety hazards for testing and deploying such a system. Testing can be managed using light-safe areas and segregated rooms, and the high gamma radiation levels in radiation areas where this technology is expected to be deployed means that human access to the areas in question is already extremely restricted or impossible to carry out safely, which makes the risks manageable for the (fully remote) human operators. Still, a laser diode using non-visible light would be advantageous, and so future work should also investigate laser diode—light receiver combinations, which match this requirement.

This work has focused on the development of a LIDAR system compatible with high radiation environments and has tackled aspects related to the theoretical radiation tolerance of the individual components and integrated subsystems, as well as the functional performance of the sensor. There are, however, other aspects of operating a LIDAR device within nuclear fusion or decommissioning environments that have not been covered within this work and would need to be suitably addressed prior to transitioning this work into a workable product. This would include challenges such as optical alignment, stability, thermal effects, and trade-offs between pulse width, intensity, and range for specific harsh environments. Depending on the exact nature of the environment, there may be further considerations to be taken including operation in vacuum conditions, magnetic fields, or with the presence of water or humid conditions.

## 8. Conclusions and Future Work

This work describes key initial steps towards de-risking a radiation-tolerant 1D LIDAR system for high gamma radiation environment applications. A novel rad-hard design using specialised design approaches was designed, implemented and the performance was tested in a lab environment. The results show that the basic principle of the LIDAR design is working, with a clear linear response over several measurement runs, which should enable a calibrated system based on the same technology in future. The material selection and overall design are expected to result in a gamma radiation total integrated dose tolerance of ∼1 MGy.

An obvious limitation of this initial study is the absence of radiation testing of the prototype system, which was out of scope of the work reported here. As a next step, gamma irradiation trials of components and subsystems used in the design will be carried out, to verify the intended radiation tolerance. Currently, no specific test data is available for most of the components selected and incorporated in the design: they are all expected to perform well under gamma dose rates of 1 kGy/h up to total doses of 1 MGy given their material composition, but this must be proven experimentally in future work. This testing will be carried out with components biased to a representative voltage level to correspond to expected active use, and multiples of each component will be tested to enable further analysis and gain confidence in the results.

Future design development is also planned once radiation testing has verified the design approach. As a first step, both the receiver and transmitter boards should be made more robust, with more compact layouts. In addition, both devices will be tested on the measurement of objects at greater ranges to investigate the applicability of the technology to a broader range of distance scenarios.

The longer term roadmap will involve implementing missing subsystems such as the ultra-short pulse generator and bias power supplies. Furthermore, the COTS rad-hard TDC described in Section 3 should be incorporated into the system and fully tested. Eventually, on-board logic functionality will need to be developed to enable a fully featured radiation tolerant LIDAR. Robotic deployment trials in real-world environments with radiation will also be conducted with further refined versions of the prototype presented here in order to further understand performance and reliability within representative operating conditions.

In conclusion, these results significantly de-risks the feasibility of long-term deployment of LIDAR systems utilising these approaches into environments with high gamma dose rates, such as nuclear fission decommissioning, big science facilities such as the Large Hadron Collider at CERN, and remote maintenance systems used in future nuclear fusion power plants such as STEP and EU-DEMO. This represents another step in the direction of increasing feasibility of remote measurements, increasing safety by removing the need for personnel deployment into radiation areas, and contributing to enabling automation of maintenance and inspection tasks.

## Figures and Tables

**Table 1:** Transmitter component table.

Functional Role	Component Selected (Manufacturer)	Material or Technology	Design Justification
Laser diode for transmission circuit	PLPT9-450LB_E (ams-OSRAM AG, Premstaetten, Austria)	InGaN	InGaN laser diodes have an expected luminosity reduction of 20% at 1.5 MGy, which is acceptable for this application.
Laser Driver, SMPS GaN FET	EPC21601 (EPC Space, Andover, MA, USA)	GaN	This driver produced by EPC uses a GaN IC and is theoretically highly gamma resistant.
NMOS Inverters	EPC8004 and EPC2037 (EPC Space, Andover, MA, USA)	GaN	FETs used to create a high slew rate edge.

**Table 2:** Receiver component table.

Functional Role	Component Selected (Manufacturer)	Material or Technology	Design Justification
MPPC (SiPM) photon detector	S14160-1310PS/S14160-1315 (Hamamatsu Photonics K.K., Hamamatsu City, Japan)	Silicon	Chosen due to expected radiation tolerance and ease of integration. Tolerance is expected into the MGy range with some compensation potentially needed.
Inverting discriminator	EPC2037 and EPC8004 (EPC Space, Andover, MA, USA)	GaN	Used to convert the optical signal into a logic signal due to extremely low input capacitance and rad-hard performance.

## Data Availability

The raw data supporting the conclusions of this article will be made available by the authors upon request.

## References

[1] Goiffon V., Rizzolo S., Corbière F., Rolando S., Bounasser S., Sergent M., Chabane A., Marcelot O., Estribeau M., Magnan P. (2018). Total Ionizing Dose Effects on a Radiation-Hardened CMOS Image Sensor Demonstrator for ITER Remote Handling. IEEE Trans. Nucl. Sci..

[2] Yang T., Hu J., Li Y., Zhao C., Sun L., Krajnik T., Yan Z. (2025). 3D ToF LiDAR for Mobile Robotics in Harsh Environments: A Review. Unmanned Syst..

[3] Zhang J., Singh S. LOAM: Lidar Odometry and Mapping in Real-time. Proceedings of the Robotics: Science and Systems (RSS ’14).

[4] Bogue R. (2022). The growing importance of LIDAR technology. Ind. Robot.

[5] Crofts O., Loving A., Torrance M., Budden S., Drumm B., Tremethick T., Chauvin D., Siuko M., Brace W., Milushev V. (2022). EU DEMO Remote Maintenance System development during the Pre-Concept Design Phase. Fusion Eng. Des..

[6] Jonasson E.T., Boeuf J., Kyberd S., Skilton R., Burroughes G., Amayo P., Collins S. (2019). Reconstructing JET using LIDAR-Vision fusion. Fusion Eng. Des..

[7] Jonasson E.T., Boeuf J., Murcutt P., Kyberd S., Skilton R. (2020). Improved reconstruction of JET using LIDAR-Vision fusion. Fusion Eng. Des..

[8] Staniaszek M., Flatscher T., Rowell J., Niu H., Liu W., You Y., Gadd M., Mattamala M., Schutz A., De Martini D. (2025). AutoInspect: Towards Long-Term Autonomous Inspection and Monitoring. IEEE Trans. Field Robot..

[9] Zhang K., Plianos A., Raimondi L., Abe F., Sugawara Y., Caliskanelli I., Cryer A., Thomas J., Pacheco-Gutierrez S., Hope C. (2025). Towards safe, efficient long-reach manipulation in nuclear decommissioning: A case study on fuel debris retrieval at Fukushima Daiichi. J. Nucl. Sci. Technol..

[10] Jonasson E.T., Ramos Pinto L., Vale A. (2021). Comparison of three key remote sensing technologies for mobile robot localization in nuclear facilities. Fusion Eng. Des..

[11] Dubus G., Puiu A., Bates P., Damiani C., Reichle R., Palmer J. (2014). Progress in the design and R&D of the ITER In-Vessel Viewing and Metrology System (IVVS). Fusion Eng. Des..

[12] Neri C., De Collibus M.F., Rossi P., Mugnaini G., Monti C., Pollastrone F., Francucci M., Fornetti G., Guarneri M., Nuvoli M. (2019). Upgraded concepts and design of an in vessel inspection system for fusion reactors. Fusion Eng. Des..

[13] Neri C., Bartolini L., Coletti A., Ferri de Collibus M., Fornetti G., Pollastrone F., Riva M., Semeraro L. (2007). The laser in vessel viewing system (IVVS) for ITER: Test results on first wall and divertor samples and new developments. Fusion Eng. Des..

[14] Cao Y., Leroux P., De Cock W., Steyaert M. Design and assessment of a 6 ps-resolution time-to-digital converter with 5 MGy gamma-dose tolerance for nuclear instrumentation. Proceedings of the 2011 2nd International Conference on Advancements in Nuclear Instrumentation, Measurement Methods and their Applications.

[15] Cao Y., Verbeeck J. (2018). MAGICS Radiation-Hardened Control System Equipment Study: Integrated Circuits for Sensing and Motor Control.

[16] Boschetto A., Bottini L., Macera L. (2023). Design and fabrication by selective laser melting of a LIDAR reflective unit using metal matrix composite material. Int. J. Adv. Manuf. Technol..

[17] Hamamatsu Photonics What Is MPPC (SiPM)?. https://www.hamamatsu.com/eu/en/product/optical-sensors/mppc/what_is_mppc.html.

[18] Makowski D. (2006). The Impact on Electronic Devices with the Special Consideration of Neutron and Gamma Radiation Monitoring. Ph.D. Thesis.

[19] Chesnevskaya S., Via C., Utting B., Hughes H., Watts S. Radiation Testing of Robotic Systems—LiDAR as a Case Study—Abstract. Proceedings of the 2019 IEEE Nuclear Science Symposium (NSS) and Medical Imaging Conference (MIC).

[20] Leroux P., Van Koeckhoven W., Verbeeck J., Van Uffelen M., Esqué S., Ranz R., Damiani C., Hamilton D. (2014). Design of a MGy radiation tolerant resolver-to-digital convertor IC for remotely operated maintenance in harsh environments. Fusion Eng. Des..

[21] Nava F., Vittone E., Vanni P., Verzellesi G., Fuochi P., Lanzieri C., Glaser M. (2003). Radiation tolerance of epitaxial silicon carbide detectors for electrons, protons and gamma-rays. Nucl. Instrum. Methods Phys. Res. Sect. A Accel. Spectrometers Detect. Assoc. Equip..

[22] Mohamed N.S., Wright N.G., Horsfall A.B. (2017). Dose Rate Linearity in 4H-SiC Schottky Diode-Based Detectors at Elevated Temperatures. IEEE Trans. Nucl. Sci..

[23] Pearton S.J., Ren F., Patrick E., Law M.E., Polyakov A.Y. (2015). Review—Ionizing Radiation Damage Effects on GaN Devices. ECS J. Solid State Sci. Technol..

[24] Ueno K., Tadokoro T., Ueno Y., Sasaki K., Koizumi S., Chayahara A., Mokuno Y., Hirano S., Kaneko J.H. (2019). Heat and radiation resistances of diamond semiconductor in gamma-ray detection. Jpn. J. Appl. Phys..

[25] Bräunig D., Wulf F., Barbottin G., Vapaille A. (1999). Chapter 10 Radiation effects in electronic components. New Insulators, Devices and Radiation Effects.

[26] Arrow Electronics Silicon vs. Gallium Nitride (GaN) Semiconductors: Comparing Properties & Applications. https://www.arrow.com/en/research-and-events/articles/gan-vs-silicon-semiconductor-materials-compared.

[27] Maset E., Martín-Holgado P., Morilla Y., Gilabert D., Sanchis-Kilders E., Martínez P.J. (2022). Temperature-Dependent Dynamic on Resistance in Gamma-Irradiated AlGaN/GaN Power HEMTs. Appl. Sci..

[28] Hazdra P., Popelka S. (2017). Radiation resistance of wide-bandgap semiconductor power transistors. Phys. Status Solidi (A).

[29] Biró B., David G., Fenyvesi A., Haggerty J.S., Kierstead J., Mannel E.J., Majoros T., Molnar J., Nagy F., Stoll S. (2019). A Comparison of the Effects of Neutron and Gamma Radiation in Silicon Photomultipliers. IEEE Trans. Nucl. Sci..

[30] Li Z., Zhou D., Xu W., Ren F., Zhang R., Zheng Y., Lu H. (2022). Demonstration of a Solar-Blind Single-Photon Imaging Lidar Based on SiC Ultraviolet Avalanche Photodiodes. IEEE Photonics Technol. Lett..

[31] Yuan P., Sudharsanan R., Bai X., Boisvert J., McDonald P., Labios E., Salisbury M.S., Stuart G.M., Danny H., Portillo A.A., Turner M.D., Kamerman G.W. (2010). 32 × 32 Geiger-mode ladar camera. Proceedings of the Laser Radar Technology and Applications XV.

